# Molecular-Genetic Portrait of Breast Cancer with Triple Negative Phenotype

**DOI:** 10.3390/cancers13215348

**Published:** 2021-10-26

**Authors:** Marina K. Ibragimova, Matvey M. Tsyganov, Nikolai V. Litviakov

**Affiliations:** 1 Cancer Research Institute, Tomsk National Research Medical Center of the Russian Academy of Sciences, 634009 Tomsk, Russia; tsyganovmm@yandex.ru (M.M.T.); nvlitv72@yandex.ru (N.V.L.); 2 National Research Tomsk State University, 634050 Tomsk, Russia

**Keywords:** triple-negative breast cancer, microarray analysis, CNA, prognosis

## Abstract

**Simple Summary:**

Breast cancer is a genetically heterogeneous disease with different molecular biological and clinical characteristics. The available knowledge about the genetic heterogeneity of the most aggressive molecular subtype of breast cancer—triple-negative—has led to discoveries in drug treatment. Identification of the molecular-genetic phenotype of breast cancer is an important prognostic factor of the disease and allows personalization of the patient’s treatment.

**Abstract:**

Understanding of the genetic mechanisms and identification of the biological markers of tumor progression that form the individual molecular phenotype of transformed cells can characterize the degree of tumor malignancy, the ability to metastasize, the hormonal sensitivity, and the effectiveness of chemotherapy, etc. Breast cancer (BC) is a genetically heterogeneous disease with different molecular biological and clinical characteristics. The available knowledge about the genetic heterogeneity of the most aggressive molecular subtype of breast cancer—triple-negative (TN)—has led to discoveries in drug treatment, including the use of DNA damaging agents (platinum and PARP inhibitors) for these tumors, as well as the use of immunotherapy. Most importantly, the ability to prescribe optimal drug treatment regimens for patients with TNBC based on knowledge of the molecular-genetic characteristics of this subtype of BC will allow the achievement of high rates of overall and disease-free survival. Thus, identification of the molecular-genetic phenotype of breast cancer is an important prognostic factor of the disease and allows personalization of the patient’s treatment.

## 1. Introduction

Breast cancer (BC) is not only a heterogeneous, solid, malignant neoplasm but also an extremely heterogeneous disease in terms of prognosis and sensitivity to antitumor therapy, which is related primarily to a variety of molecular-genetic changes leading to its development and progression. Molecular classification divides breast cancer into several subtypes that differ in both malignancy and prognosis and, accordingly, sensitivity to various types of treatment [1].

There were four different molecular subtypes of BC: two subgroups with positive expression of estrogen and/or progesterone receptors (ER+/PR+)—luminal A and B, which account for approximately 70% of all breast cancer cases; one subgroup with overexpression/amplification of the HER/2-neu gene and negative expression of hormonal receptors (HER/2 positive subgroup); and triple-negative breast cancer (TNBC), with no expression of steroid hormone receptors and *HER2*. Sometimes a fifth subgroup is identified, termed unclassified breast cancer [1].

Among all molecular subtypes, breast cancer with a triple-negative phenotype represents approximately 10–24% of cases and has the most aggressive course; as a result, it has the worst prognosis [2,3]. The clinical picture of this subtype of BC is as follows: age 35–55 years, high risk of developing visceral metastasis (lungs (*p* = 0.01), brain (*p* = 0.035)), the peak of distant metastasis occurs 1–3 years after surgery treatment, and survival rates are lower than other subtypes [4]. At the same time, for these patients, a high proliferative index (average Ki67 values more than 40%), and the *BRCA1* gene mutation are often detected [5].

The literature describes atypical factors for this subtype of breast cancer. Patients with TNBC are more sensitive to neoadjuvant chemotherapy (NAC) and achieve good treatment results in the form of a complete pathological response. If a complete pathological response is not achieved, the best result of preoperative treatment is combined with a worse prognosis in the postoperative period, lower rates of relapse-free and overall survival (*p* < 0.0001), and a higher probability of disease relapse. This phenomenon is called the triple-negative breast cancer paradox [6,7]. A huge amount of research is devoted to the search for approaches to the treatment of TNBC [8,9,10,11,12,13,14,15,16,17,18]. Currently, chemotherapy, alone or in combination with surgery and/or radiation therapy, is the standard treatment for TNBC. The main problem of targeted therapy for TNBC is the absence of specific oncogenic factors due to its wide heterogeneity. According to the recommendations of ST. Gallen-2015 and ESMO-2015, patients with this molecular subtype are subject to chemotherapy with taxanes and anthracyclines, and in the presence of a BRCA mutation, it is also possible to use platinum drugs [19].

The reasons for this TNBC paradox are still unclear. However, it should be noted that patient selection based on molecular-genetic biomarkers at the initial stage of treatment can improve the effectiveness of treatments for this aggressive BC subtype. There is no doubt that a better understanding of the nature of TNBC will allow the use of individual and more effective methods of treatment. However, there are no clearly defined prognostic criteria for predicting the course of TN breast cancer. It is important to systematize and verify both the available literature data and the search for molecular-genetic features for a given molecular subtype to identify new markers of the course and outcome of the disease.

More than 10 years ago, Mersin et al. found that patients with TNBC had a higher risk of recurrence, and the mean time to relapse was over 1 year, which was shorter than that in other BC patients [20]. The search for markers to predict the course of the disease, as well as the search for an approach to the prevention of relapses and metastases, is critical for improving the survival of patients with TNBC [21]. To solve this problem, it is necessary to understand the molecular-genetic mechanisms characteristic of TN breast cancer, which can serve as a basis for searching for potential molecular-genetic markers of the course of the disease and predicting the outcome of patients with triple-negative breast cancer.

The presented review is devoted to the analysis of the most relevant data of the molecular-genetic parameters of TN breast cancer from the point of view of their possible use as targets for therapy.

## 2. Molecular-Genetic Mechanisms Presenting TNBC

A total of 1.68 somatic mutations per Mb of coding regions (~60 somatic mutations in each tumor) have been described for TNBC. The frequency of mutations is different for each tumor, and some have a high mutation load of more than 4.68 somatic mutations per MB [22,23,24] and the frequent occurrence of multiple copy number aberrations involving genes that lead to changes in the corresponding cellular pathways. According to the literature, there are several genes with a high frequency of occurrence of the aberrant state. *TP53*, in first place, has an average mutation rate of 60–70% in TN breast cancer [22,25]. Approximately the same frequency of occurrence (10%) is observed for *BRCA1/2*, whose mutation can increase mortality up to 60–70%, and for *PIK3CA* [25,26]. Table 1 shows the main genes that can be mutated in TNBC and their cell-signaling pathways (according to the literature data) [25,27,28,29,30].

**Figure 1:**
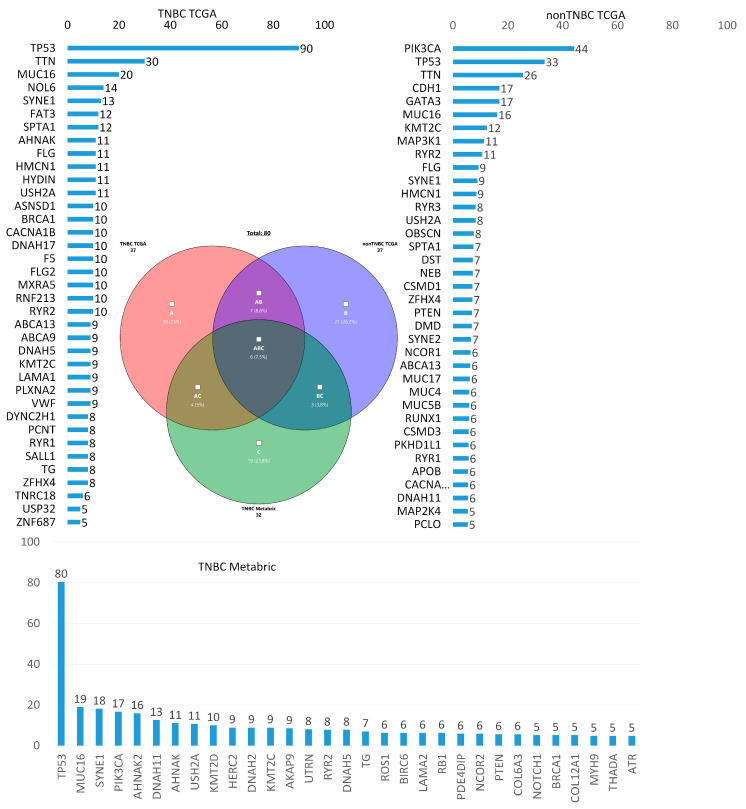
Frequencies of mutations in tumors of patients with TNBC from TCGA, non-TNBC TCGA, and TNBC Metabric.

According to TCGA, the frequency of tumor gene mutations was assessed in 171 patients with TNBC and in 774 patients with breast cancer of other molecular subtypes (non-TNBC). The Metabric database (270 patients with TNBC) was also used for additional analysis. Figure 1 shows the genes whose somatic mutations are most common in TNBC in comparison with non-TNBC according to TCGA database and the frequency of gene mutations according to the Metabric database. The following is a Venn diagram that shows genes common and unique to TNBC and non-TNBC. Thirteen genes were common to TNBC and non-TNBC (*HMCN1*, *RYR2*, *FLG*, *SPTA1*, *USH2A*, *TTN*, *SYNE1*, *ABCA13*, *KMT2C*, *ZFHX4*, *TP53*, *RYR1*, *MUC16*) and 24 genes were unique to TNBC (*PLXNA2*, *ASNSD1*, *F5*, *FLG2*, *ZNF687*, *CACNA1B*, *TG*, *DYNC2H1*, *FAT3*, *RNF213*, *DNAH17*, *HYDIN*, *ABCA9*, *AHNAK*, *USP32*, *SALL1*, *PCNT*, *BRCA1*, *NOL6*, *DNAH5*, *LAMA1*, *VWF*, *TNRC18*, *MXRA5*). These were somatic genes with a frequency of more than 5%. Despite the fact that the *TP53* gene is most often mutated in TNBC and is also common to TNBC and non-TNBC, according to TCGA, the frequency of *TP53* gene mutations in TNBC tumors is almost three times higher than in non-TNBC patients, while the nonsense, Frame_Shift_Del, and Frame_Shift_Ins mutations are almost half as high (44.0%). The pathways characteristic of these unique genes are listed in Table 1.

Only TNBC somatic mutations were compared according to TCGA and Metabric databases. The bases overlap in four genes (*DNAH5*, *TG*, *AHNAK*, and *BRCA1*). This low level of database overlap is due to the outdated nature of the Metabric database, in which only 155 targeted genes are sequenced, as opposed to the genome-wide sequencing of TCGA. The pathways characteristic of these four genes are highlighted in blue in Table 2.

The *HYDIN* (16q22.2), *ASNSD1* (2q32.2), *DNAH17* (17q25.3), *MXRA5* (Xp22.33), *TNRC18* (7p22.1), *USP32* (17q23.3), and *ZNF687* (1q21.3) signaling pathways are not yet known.

In the next stage, the cell-signaling pathways characteristic of TNBC and non-TNBC were analyzed. Supplementary Tables S1 and S2 present data on cellular signaling pathways characteristic of TNBC and non-TNBC according to the TCGA database. Thus, 50 cellular signaling pathways characteristic of breast cancer with a triple-negative phenotype (Supplementary Table S1), 200 cellular signaling pathways characteristic of other molecular subtypes of breast cancer, and 79 common cellular signaling pathways (Table 2, Supplementary Materials) were identified.

**Figure 2:**
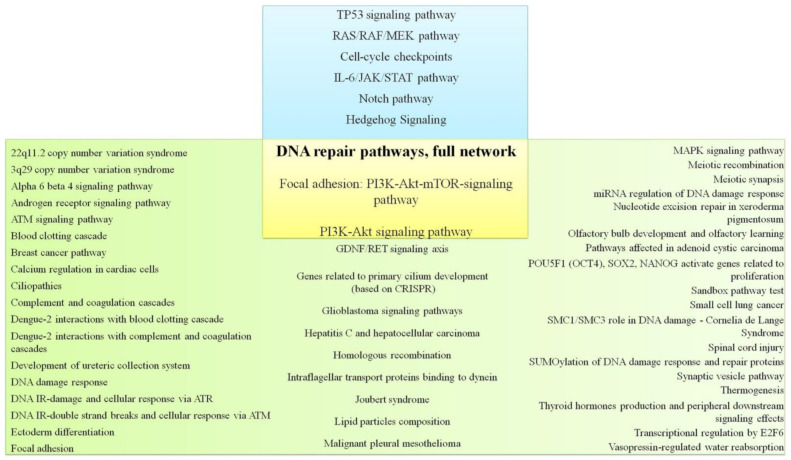
Signaling pathways for TNBC according to the literature data and signaling pathways determined by genes with mutations that are found in TNBC but not in non-TNBC according to TCGA database.

When correlating the literature data on the cell-signaling pathways and pathways characteristic of TNBC, determined by genes whose mutations occur in TNBC and not in non-TNBC according to TCGA database, the intersection of three cellular pathways was revealed—DNA repair pathways, focal adhesion: PI3K-Akt-mTOR-signaling pathway, and PI3K-Akt signaling pathway (Figure 2). Obviously, it makes sense to pay closer attention to these paths. Separately, it should be noted that DNA repair pathways are determined according to TCGA, Metabric, and literature data.

### 2.1. DNA Repair Pathway

Chemotherapy is the main treatment for TNBC, using drugs that damage the DNA of the tumor. Repairing double-stranded DNA breaks weakens the effects of chemotherapy and allows tumor cells to resist DNA damage by chemotherapy [31]. Lin et al. showed that IGFBP-3 protein can form a nuclear complex with *EGFR* and DNA-dependent protein kinases, and this complex repairs double-stranded DNA breaks with NHEJ in TNBC cells [32]. Another study showed that an octamer-binding protein lacking the POU domain and its dimerization splicing partner, proline/glutamine-rich protein, can form a PARP-dependent complex with IGFBP-3, and DNA damage is inhibited by this complex after chemotherapy. The possibility of reducing therapeutic TN breast cancer cell resistance with a subsequent improvement in the therapeutic effect is assumed [33].

There are also many studies in the literature in which the expression profiles of a number of genes involved in various pathways of DNA repair were studied in TNBC as surrogate markers of the DNA repair status. E. Ribeiro et al. studied the expression of 13 DNA repair genes in TNBC (*PARP1*, *ERCC1*, *XPA*, *XPD*, *XPF*, *XPG*, *BRCA1*, *FANCA*, *FANCC*, *FANCD2*, *FANCF*, *PALB2*, and *Chk1*) compared with breast cancer of the luminal A subtype. It was shown that the expression of most genes, nucleotide excisional repair, was significantly reduced in TNBC. At the same time, the level of *PARP1* expression was higher in TNBC, and a high level of *FANCA* correlated with increased overall and disease-free survival in TNBC [34].

Additionally, interesting data were published by Marie Ollier and colleagues. In this work, using NGS, 36 candidate genes that were known or assumed to be involved in the process of DNA repair were analyzed (*POLB*, *ERCC1*, *APC*, *EPCAM*, *MLH1*, *MLH3*, *MSH2*, *MSH6*, *MUTYH*, *PMS1*, *PMS2*, *PTEN*, *TP53*, *ATM*, *AURKA*, *CHEK2*, *CDH1*, *NRIP1*, *FGFR2*, *STK11*, *WRN*, *BRCA1*, *BRIP1*, *BARD1*, *COBRA1*, *BRAP*, *BRCC3*, *PALB2*, *BAP1*, *BLM*, *MRE11A*, *NBN*, *RAD50*, *RADC51D*, and *RAD51*) for 50 patients with TNBC without BRCA1/BRCA2 mutations. Seven missense mutations were found in 50 patients in six different genes: *RAD51D* (two cases), *MRE11A*, *CHEK2*, *MLH1*, *PALB2*, and *MSH6.* The authors proved that the presence of mutations in the *RAD51D*, *MRE11*, and *PALB2* genes creates the risk of developing TN breast cancer [35].

### 2.2. TP53 Signaling Pathway

*TP53* mutations are extremely common in TN breast cancer, which potentially makes the TP53 signaling pathway a suitable target for targeted treatment of this subtype [36]. Cell apoptosis is initiated by p53 if there are a large number of DNA mutations or chromosomal damage. MTP53 acts as an oncogene and promotes the transformation of normal cells into tumor cells. When WTP53 mutates into MTP53, it is often inactivated, which leads to cell carcinization and excessive proliferation, ultimately leading to tumorigenesis. In TNBC, mutations inactivating TP53 are observed in almost half of the patients (see above). In addition, TP53 is often inactivated by deletion of the locus in triple-negative breast cancer. *TP53* interacts directly with several proteins, including *CDKN1A*, *SFN*, *EI24*, *SERPINE1*, *DDB2*, *STEAP3*, and *MDM2*. The interaction between p53 and MDM2 plays an important role in the regulation of the cell cycle. p53 is transferred from the nucleus to the cytoplasm using *MDM2* and then degrades. The *MDM2* level is regulated by p53, and a negative feedback loop is formed between *MDM2* and p53. This keeps the *MDM2* to p53 ratio constant [37]. In 2015, Sun et al. reviewed progress in targeting p53 in dynamics and mathematical models, with a proposal for a new cancer target [38]. A review by Turner and colleagues looked at several agents with potential activity against aberrant p53 signaling as a novel approach to finding effective targeted therapies for this aggressive subtype of breast cancer. There is no answer regarding whether targeted therapy against the mt-p53 pathway can be successfully used in TNBC [36].

### 2.3. PI3K/AKT/mTOR Pathway

Oncogenic activation of the PI3K/AKT/mTOR pathway may occur as a function of overexpression of upstream regulators (e.g., *EGFR*), activation of *PI3Kα* catalytic subunit (*PIK3CA*) mutations, loss of function, or *PTEN* expression in TNBC [39,40]. In contrast, activating mutations of downstream molecules with respect to PI3K (i.e., AKT and mTOR) and related pathways (*MAPK* and *RAS*) are rare for TNBC. Moreover, several oncogenic pathways (e.g., *FGFR*, cMET, and *RAF*) caused by inactivation of P53 interact to activate the PI3K pathway [41]. Deng et al. found that the combination of SF1126 (a PI3K inhibitor) and gefitinib significantly facilitates the apoptosis of TNBC cells in a dose-dependent manner [42]. Other researchers have found that disabling AKT3 significantly reduces TNBC cell proliferation in vitro and in xenografts. The size of the xenograft tumors also decreased with the addition of the AKT inhibitor GSK690693 in vivo [43]. Ueng and colleagues found that 72.1% of 172 cases of TNBC were positive for phosphorylated p-mTOR and that p-mTOR expression was associated with worse overall and disease-free survival in early TNBC [44].

### 2.4. RAS/MAPK, RAS/RAF/MEK Pathways

A number of hypotheses have been proposed about the mechanisms of alternative or noncanonical activation of the MAPK pathway. Overexpression of RAS proteins was found in approximately 30% of cancers and is used as a potential target for treatment [45]. De Luca and colleagues showed that the RAF/MEK/ERK signaling pathway plays an important role in breast cancer and can regulate cell growth and survival [46]. It was also shown that the RAF/MEK/ERK pathway was activated in 14 TNBC samples, and the authors suggested that MEK could be used as a molecular target for the treatment of this subtype [47]. Balko et al. used NanoString analysis to study gene expression patterns in 49 residual breast cancer tumors after neoadjuvant chemotherapy and showed that *DUSP4* is a negative regulator of ERK [48]. Then, they analyzed 89 samples of TNBC after NAC and showed that the level of *DUSP4* mRNA was the lowest in cell lines and tumors. The authors concluded that DUSP4 suppression activates the RAS/ERK pathway, which leads to a decrease in tumor cell sensitivity to chemotherapy [49].

Thus, copy number changes in the components of the canonical RAS/MAPK pathway (i.e., amplification of *KRAS* and *BRAF*) are thought to be associated with TNBC [47]. However, the biological consequences of increasing the copy number of the wild-type allele (as opposed to mutational activation) are not fully understood. In addition, TNBC is known to have more copy number changes than other subtypes. Therefore, the association of amplification of these components can be a marker of genomic instability and not ascribe a true oncogenic function [22].

### 2.5. Cell-Cycle Checkpoints

*CHEK2* (cell-cycle-checkpoint kinase 2) is another important gene for breast cancer susceptibility, discovered after BRCA1/2. The literature reports on the critical role of *CHEK2* in the regulation of apoptosis, the cell cycle, and DNA repair [50].

Heijink et al. described a systematic approach to identify molecular signals that can differentiate cisplatin-sensitive from cisplatin-resistant TNBC cell lines. The authors found that cell-cycle checkpoint factors appear to determine susceptibility to cisplatin in TNBC cell line models that do not have DNA repair defects [51]. These results are consistent with earlier observations that *WEE1* and *CHK1* expression levels are associated with cisplatin sensitivity [52], and targeting cell-cycle checkpoints, including *ATR*, *CHK1,* and *WEE1*, can be used to sensitize tumor cells to cisplatin.

Li Luo et al. studied the mechanism of *CHEK2* kinase gene dysfunction in TNBC cells resistant to chemotherapeutic drugs. It has been shown that the *CHEK2* Y390C mutation induces drug resistance in MDA-MB-231 cells and disrupts the inhibition of cell viability (blocking p53 activation) upon DNA damage caused by exposure to cisplatin [53].

CDK4/6 inhibitors effectively suppress the proliferation of TNBC cells with Rb protein expression. Moreover, loss-of-function Rb mutations result in resistance to CDK4/6 inhibitors. In turn, deletion of *Rb1* can lead to increased expression of p16ink4a. Loss of p16ink4a’s function as a tumor suppressor may confer high sensitivity to CDK4/6 inhibitors. The reasons for the loss of Rb and the mechanisms of overexpression of p16ink4a are unclear, but p16ink4a and Rb may be important biomarkers for predicting the response to CDK4/6 inhibition in TNBC [54,55,56]. A recent study by Ye Hu and colleagues also analyzed the potential for the use of CDK4/6 inhibitors in malignant breast cancer. The authors have shown that CDK4/6 inhibitors can improve the effectiveness of other drugs, especially chemotherapy, which remains the standard treatment for this subtype of breast cancer due to its extremely aggressive nature and lack of established molecular targets for therapy. Genetic mutations also confer resistance of tumor cells to CDK4/6 inhibitors, including mutations in *TP53* and *MYC*. However, further research is needed to identify additional potential targets that could predict the efficacy of CDK4/6 inhibitors in malignant breast cancer [57].

### 2.6. IL-6/JAK/STAT Pathway

In addition to the roles that each STAT-signaling protein may play in promoting or inhibiting tumorigenesis, additional roles that individual proteins in this family play when coactivated are just beginning to be explored. For example, the *STAT1*, *STAT3,* and *STAT5* proteins are simultaneously phosphorylated in a number of human neoplastic pathologies, including breast, lung, head, and neck cancers. For example, a functional interaction between activated forms of *STAT3* and *STAT5* has been described in breast cancer. Activated *STAT3* and IL-6 are found in TN breast cancer or high-grade tumors and are associated with poor response to chemotherapy [58]. Prolactin-mediated *STAT5* activation in breast cancer can lead to repression of BCL6 transcription, the overexpression of which is associated with the development of a metastatic form of this oncopathology [59]. *JAK1/2* inhibition reduces the number of CD11b/Gr1-positive cells in the TH mouse model of breast cancer, and although the effect on T-cell subpopulations/activity has not been reported, the authors conclude that they will have a significant effect [60].

### 2.7. NOTCH Pathway

*NOTCH* signaling is often disrupted in breast cancer, and *Notch* hyperactivation contributes to the tumor process. Gene rearrangements that generate versions of Notch receptors to enhance their function have long been described in breast tumors [61]. Upregulation of Notch signaling is associated with loss or inactivation of the Numb-negative Notch regulator [62]. Similarly, increased expression of the Notch ligand JAGGED1 correlates with poor prognosis in breast cancer, and overexpression of *NOTCH1* and *NOTCH4* receptors is observed in TNBCs [63]. Tumor stem cells (CSCs) also play an important role in the initiation and metastasis of breast cancer [64]. Notch1 affects breast CSC self-renewal by increasing ErbB2 transcription [65]. At the same time, a comparison of activated Notch receptors in CSCs and tumor-initiating mammary cells showed that Notch4 is activated in TNBC CSCs [66]. TNBC cells ectopically expressing Notch4 showed increased proliferation and invasiveness, while inhibition/knockdown of Notch4 decreased cell proliferation, invasion, tumor volume, and tumorigenicity [67].

Wang et al. showed that the NOTCH pathway can be activated after mutations in the *NOTCH1*, *NOTCH2*, and *NOTCH3* PEST domains. Approximately 13% of TNBC has mutations in the PEST domain and cause activation of pathways. Therefore, these mutations can be used as biomarkers for screening patients who may be sensitive to NOTCH inhibitors [68].

Moreover, in TNBC, for example, the antitumor effect of oridonin was reported for the first time both in vitro (cell line 4T1) and in vivo with inhibition of *NOTCH 1–4* expression [69].

### 2.8. Hedgehog Signaling

Extensive preclinical studies have highlighted the key contribution of Hh signaling to tumor stem cell reprogramming in malignant breast cancer [70,71]. The role of Hh signaling was shown in the regulation of stem cell factor BMI-1 and the ability of cells to form mammary spheres [72]. At the same time, suppression of mammosphere formation and a decrease in *ALDH1* in TNBC cell activity are observed when the Hh pathway is inhibited [71,73]. The role of Hh signaling in the regulation of epithelial–mesenchymal transition (EMT) has been described in several malignancies, including ovarian cancer [74], prostate cancer [75], lung cancer [76], and breast cancer, particularly the triple-negative subtype [77].

Mesenchymal marker expression and EMT factor transcription in TNBC cells are directly regulated by the Hh effector *GLI1*, and the cancellation of Hh signals leads to the loss of the mesenchymal phenotype and restores E-cadherin and keratin expression of epithelial markers [78]. In vivo mouse studies on the mammary glands illustrate that constitutive Hh signaling marked ductal hyperplasia and dysplasia caused by an increase in cell populations showing expression of basal cytokeratin P63, as well as a loss of basolateral polarity resembling EMF [79].

## 3. Molecular Markers for Immunotherapy of TNBC

Several key characteristics make TNBC more likely to respond to immunotherapy than other BC subtypes. First, TNBC has more tumor-infiltrating lymphocytes (TILs), which correlate with an improved prognosis, especially in the early stage of TNBC [80,81,82]. TILs are a predictive biomarker, and histopathological scoring has been successfully standardized by the global TILs International Working Group (TILs Working Group; available at: https://www.tilsinbreastcancer.org, accessed date 20 September 2021) [83]. Second, TNBC has higher levels of PD-L1 expression in tumor and immune cells (according to The Cancer Genome Atlas, it has been confirmed that TNBC has a higher expression of PD-L1 mRNA) [84,85], providing a direct response to anti-PD-1 therapy. TNBC has a greater number of nonsynonymous mutations that lead to the appearance of tumor-specific antigens that activate specific T cells to create an antitumor immune response, which can be enhanced by immune checkpoint inhibitors [86]. It has also been shown that TNBC has higher rates of CD8+ T lymphocyte infiltration [85,87]. A number of immune checkpoint genes were upregulated in TNBC compared with non-TNBC: *CTLA4*, *PD1*, *PD-L1*, *PD-L2*, *LAG3*, *IDO1/2*, and *TIGIT*. Of these, *CTLA4*, *PD1*, *LAG3*, *IDO1/2*, and *TIGIT* also had upregulated TNBC compared with normal tissue. In addition, TNBC has been shown to exhibit the strongest immunogenicity among the BC subtypes, thus justifying the choice of immunotherapeutic option for TNBC [88].

To date, various immunotherapeutic antibodies for TNBC targeting PD-L1 and PD-1 are actively being investigated—atezolizumab, durvalumab, avelumab, nivolumab, and pembrolizumab [89]. In addition, tremelimumab, a monoclonal antibody targeting CTLA-4, is currently being investigated in patients with TNBC [90].

Another marker of sensitivity to immunotherapy, microsatellite instability (MSI), was studied retrospectively in 440 TNBC patients. The prevalence of mismatch repair (MMR)-deficient and high-frequency MSI was found to be extremely low in TNBC at only 14%. MMR/MSI status has limited predictive value for TNBC [91]. An even lower frequency of high-frequency MSI (0.9%) was found in Japanese patients with TNBC [92]. Another marker, mutational load (TMB—tumor mutational burden), showed significant predictive value for achieving pCR in TNBC patients in a recent neoadjuvant study of immune checkpoint blockers (GeparNuevo) [93]. The prevalence of TMB ≥ 10 mut/Mb in patients with metastatic TNBC was ~10% and positively correlated with the response to pembrolizumab [94]. In general, TNBC carries a higher level of TMB than other molecular BC subtypes, and the effectiveness of immunotherapy, including in combination with chemotherapy for TNBC, is significantly higher (GeparNuevo, IMpassion130, KEYNOTE-119, TAPUR) [95].

## 4. CNA and TN Breast Cancer

There are many studies of copy number aberrations (CNA) in breast cancer in different contexts, including during treatment. However, a small amount of information is presented in the triple-negative subtype.

The most common CNAs in malignant breast cancer have been described. In 2019, Gómez-Miragaya J. and colleagues described in detail the CNA landscape of the cell model of metastatic triple-negative breast cancer. Analysis of the CNA frequency in this cell model showed the presence of the most frequent amplifications at loci 1q, 8q, and 10p, as well as frequent deletions at loci 1p, 4p, 5q, 10q, 15q, and Xp [96].

Interesting results were also obtained in the study of the frequency of chromosomal aberrations in patients with triple-negative breast cancer. Burstein and coauthors, on a large sample of patients (*n* = 278) with TNBC, characterized the features of the CNA frequency and showed that the highest amplification frequency (more than 84%) was found in the long arms of chromosomes 1, 1q31.2, 3q26.1, and 8q23.3 loci, with the highest frequency of deletions at 8p23.2, 9p21.3, and 10q23.31 loci [97].

A unique work about copy number evolution in triple-negative breast cancer was published in 2016. The authors sequenced 1000 individual cells from tumors of 12 patients and identified one to three major clonal subpopulations in each tumor that had a common evolutionary lineage for each CNA. According to the work results, amplification of chromosome 5 was common, with an increase in the number of copies of the *MAP3K1*, *ERBB2IP*, and *PIK3R1* genes, as well as 10p and 12q, with an increase in the number of copies of the GATA3 and *MDM2* genes, respectively. Moreover, we analyzed the data on six unique metastable tumor cells with nonclonal CNA amplifications of 5p and 18p. The authors also noticed that metastable tumor cells acquired a single CNA at later stages of tumor development. In addition to overgrowth of stable clonal tumor expansions, patients with triple negative breast cancer may continue to acquire a single CNA at later stages of tumor development. Nevertheless, the results show that most copy number aberrations are acquired at the earliest stages of tumor development, followed by a stable expansion of the clonal composition [98]. In another study, CNAs were found in TNBC, including amplifications at loci 1q, 8q, and 10p, deletions at loci 5q and 8p, amplifications of the *EGFR* and *FGFR2* genes, and *PARK2* and *PTEN* gene deletions [25]. The simultaneous presence of 1q amplification and 16q deletion, as in ER-positive breast cancer and triple-negative breast cancer, is not recorded [27].

Balko et al. presented molecular profiling results for triple-negative breast cancer after NAC. Evaluation of mutational alleles and CNAs showed de novo amplifications (not found in the pretreatment sample) and enrichment (found in the pretreatment sample but increased in the posttreatment sample) in the evaluation of mutational alleles and CNAs. Many of these de novo enrichments and amplifications occurred in genes including cell-cycle regulators and PI3K/mTOR pathway genes. The authors found that the number of copies of the AKT and CCND member families was increased during the NAC process. The number of MYC and MCL1 copies has been increased on several occasions after NAC. The authors note that *MCL1* amplifications may be associated with de novo resistance to chemotherapy. Amplifications of the *MCL1* (54%) and *MYC* (35%) genes were detected after NAC. Additionally, the frequency of *PTEN* deletions and *JAK2* amplifications increased in the residual tumor. These de novo acquisitions may play a role in acquired therapeutic resistance. Thus, the *CCND1*, *CCND2*, *CCND3*, *CDK4*, *IGF1R*, *PIK3CA*, *RAF1*, *JAK2*, *MDM2*, *MYC*, and *MCL1* genes were amplified, and the *AKT2* and *PTEN* genes were deleted. Many of the amplified genes belong to the group of cell-cycle regulators [48]. This study is devoted to the evolution of chemoresistance in malignant breast cancer. Exome sequencing was performed on samples from 20 patients, and more detailed analysis using single-cell DNA and RNA sequencing was performed on eight patients. Ten patients showed no mutations after treatment, while 10 showed residual mutations after treatment among 20 patients. The authors did not observe an increase in the mutational load in response to NAC in any of the patients. Sequencing was performed to investigate the evolution of copy number during NAC on 900 individual cells from the corresponding samples of eight patients—four patients with clonal extinction and four patients with clonal persistence based on their classification according to exome sequencing data. It was shown that polyclonal tumors had common evolutionary ancestors in the first group of patients, as evidenced by the common CNA of the *MET*, *MYC*, and *PTEN* genes in P6; *MDM4*, *EGFR,* and *PTEN* in P2; *MYC* and *PTEN* in P9; and *MYC*, *MET*, *TP53,* and *CDKN2A* in P1. These tumors also had CNAs that appeared at a later stage in tumor evolution. However, regardless of the clonal subpopulation number, NAC led to the disappearance of tumor cells in these patients. Furthermore, the evolution of the copy number was assessed in four patients with clonal persistence (Group 2). A smaller portion of tumor cells before treatment were grouped with tumor cells after treatment in all four patients. This suggests that they may have a stable genotype. The authors calculated consistent copy number profiles from single cells to identify specific CNAs in resistance-related clones. Although most CNAs were distributed between subclones, specific CNAs were also identified that were found exclusively in chemoresistant clones. The resistant clone had two focal deletions at 3p, including a 3p26 (IL5RA) and 3p24-22 deletion at (RARB) in sample P14. This clone expanded after NAC from 7.7% to 71.8%. In P11, two clones associated with resistance, clone C, which increased from 5.7% to 41.4%, and clone E, which appeared in the middle of treatment by 2.6% and expanded to 37.8%, appeared after NAC. Resistance-associated clone C had deletions of 4p15 (*PCDH7*, *DTHD1*), 11q21-22 (*MAML2*), and 13q (*RB1*, *BRCA2*, *FOXO1*). In contrast, the resistant clone E had deletions of 6q and 20chr (*PAK7*). The expansion of two minor clones with different genotypes suggests convergent evolution towards a stable phenotype. CNAs specific for resistant subclones were also identified at P12 and P15. However, the data did not reveal relapse of CNA in resistant clones among four patients in the second group. Additionally, 59–275 differentially expressed genes (DEGs) were identified, which were upregulated in chemoresistant tumor cells after treatment in each patient using differential expression analysis. The list includes the known genes MYC, ERBB3, KIT, and PIK3R1 [99].

## 5. Own Research

### Ethics Approval and Consent to Participate

All procedures performed in studies involving human participants were in accordance with the ethical standards of the institutional and/or national research committee and with the 1964 Helsinki declaration and its later amendments or comparable ethical standards. The study was conducted with permission by the local Ethics Committee of the Cancer Research Institute Tomsk NMRC (Protocol 1 from 14 January 2013). All patients gave their informed consent. The experimental protocols were approved by the institutional committee of Tomsk National Research Medical Center of the Russian Academy of Sciences (Protocol 3 from 16 January 2013).

**Figure 3:**
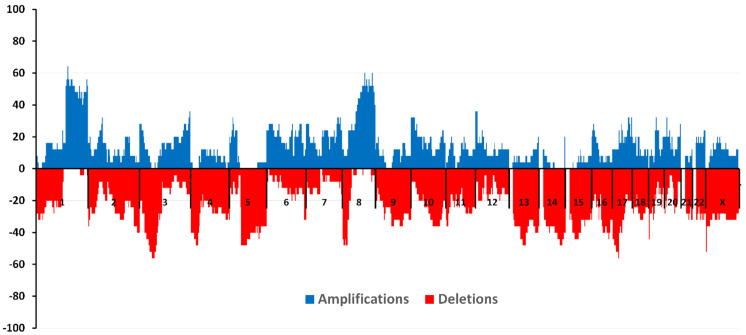
Frequencies of deletions and amplifications in triple-negative breast cancer patients.

The study included 25 patients with a diagnosis of triple-negative breast cancer. A high-density microarray CytoScan HD Array (Affymetrix, Santa Clara, CA, USA) was used to study CNA. Figure 3 shows the amplification and deletion frequencies on each chromosome for patients included in the study.

The largest number of amplifications (more than 64.0%) was found in the 1q21.3 locus in the absence of deletions in this region. This locus contains the *ZNF687* gene, the mutations of which are unique to TNBC according to TCGA data. It was shown that overexpression of *ZNF687* significantly increased the expression of pluripotency-related factors *BMI1*, *OCT4,* and *NANOG* and increased the ability of hepatocellular carcinoma cells to form tumor spheres and invade [100].

The highest frequency of deletions (more than 56.0%) was found in loci 3p21.31-21.1 (taking into account the complete absence of amplifications) and 17q11.2. The 17p13.1 locus, where the *TP53* gene is located, was deleted in 44% of patients. The following loci with simultaneous absence of segmental CNA were found—13p13-11.1, 14p13, 14p12-11.1, 14q11.1, 15p13-11.1, 15q11.1, 21p13-11.1, 21q11.1, and 22p13-11.1.

We also studied the association of NAC response with the incidence of CNA before treatment. There were two groups of patients: a group with an objective response (1)—patients with partial and complete tumor regression after treatment (*n* = 15) and a group with no response (2)—patients with stabilization and progression after NAC (*n* = 10). The groups did not differ statistically by main clinical and morphological parameters.

The maximum frequency of amplifications (80.0% or more) was found in the 1q21.3 region for the first group of patients. The complete absence of deleted regions was shown at the maximum frequency of amplifications in this region. The frequency of amplified sites did not exceed 40% in patients with no NAC response. The maximum number of deletions (66.67% or more) was noted in the 17q11.2 locus without amplifications at this locus in Group 1 of patients.

For Group 2 patients, it was found that the largest number of amplifications (more than 60.0%) against the complete absence of deleted sites was found in loci 5p15.1 and 8q22.3, while for patients with an objective NAC response in this region, the amplification frequency did not exceed 13.3%. The maximum number of deletions (more than 70.0%) against the complete absence of amplified regions in this group of patients was found in the short arm of chromosome 3 at locus 3p21.31-21.1.

Comparison of the CNA frequencies in these groups of patients using the two-sided Fisher test showed that the objective response to NAC was observed with a greater number of amplifications in the 3q23 region. Amplification was identified in 30.0% of patients with partial and complete regression compared with patients with stabilization and progression (*p* = 0.03). Potentially, this locus can be considered a predictive marker of a good NAC response in patients with TNBC.

## 6. CNA and TNBC: Relationship with Hematogenous Metastasis

TNBC is an aggressive metastatic subtype of breast cancer, with 35% of patients showing metastases five years after diagnosis [101]. In our study, the incidence of hematogenous metastasis was 16.0%. The association of the hematogenous metastasis status of patients with the CNA incidence before treatment was investigated. Two groups of patients were identified: group (1)—with the presence of hematogenous metastasis (*n* = 4) and group (2)—with the absence of hematogenous metastasis (*n* = 21).

The maximum frequency of occurrence of amplifications (75.0% or more) was found in the short arm of chromosome 5 (region 5p14.3-13.2) for the first group of patients. The complete absence of deleted regions was shown at the maximum frequency of amplifications in this region. In patients with the absence of hematogenous metastasis in these regions, the frequency of amplified sites did not exceed 19.0%. The maximum number of deletions (70.0% or more) (with no amplifications) in Group 1 patients was observed in the 4q24-28.3 and 22q12.3 loci.

For the second group of patients, it was found that the largest number of amplifications (more than 66.67%) against the complete absence of deleted regions was found in the 1q21.3 locus. The maximum number of deletions (more than 57.1%) against the complete absence of amplified regions in this group of patients was found in the Xp22.33 region. It is noteworthy that this locus contains the *MXRA5* gene, mutated in TNBC and for which the pathways are unknown. Low expression of this gene in lung adenocarcinoma tissue was associated with better survival rates [102]. High expression of *MXRA5* has also been identified as a marker of poor prognosis in colorectal cancer [103]. These results are consistent with our data on the association of deletions of the *MXRA5* gene locus with a favorable outcome.

As a result of comparing the CNA frequencies in these groups of patients using the two-tailed Fisher test, it was shown that the presence of hematogenous metastasis was observed with a greater number of amplifications in the 5p14.2 region (*p* = 0.018) and with a greater number of deleted sites in the 4q26 region (*p* = 0.04).

## 7. Conclusions

It has been established that TNBC is a very aggressive, highly metastatic, and very complex subtype of breast cancer, for which there are no specific targets or targeted therapeutic agents. Due to molecular heterogeneity, high malignancy, lack of molecular targets, rapid metastasis, and sensitivity to chemotherapy, there is no targeted therapy to treat and prevent relapse in TNBC.

Recently, the development and widespread use of genomics, epigenomics, transcriptomics, and proteomics technologies has allowed us to take a fresh look at the molecular complexity of this disease [101]. In this work, we consider recent progress in the molecular-genetic study parameters of TNBC as a potential therapeutic target for treatment.

Our own data partially agree with the previously presented results; in particular, the highest frequency of amplifications is also noted in the long arm of chromosome 1 [96,97].

However, fundamentally new results were obtained regarding the genetic NAC response of triple-negative breast tumors and the relationship with distant metastasis. It is important to understand that such data, taken together, can form the basis for the development of new markers of treatment efficacy in patients with triple-negative breast pathology.

## Figures and Tables

**Table 1:** Cell-signaling pathways in TNBC.

	Aberrant Gene States	Pathway
1	*BRCA1/2* mut/del	DNA repair pathway
2	*CDKN1A*, *SFN*, *EI24*, *SERPINE1*, *DDB2*, *STEAP3*, *MDM2* over-exp	TP53 signaling pathway
3	*PIK3CA* mut/amp; *AKT3* amp/mut; *PTEN* del/mut; *TSC1* del/mut; *INPP4B* del; *TSC1*	PI3K/AKT/mTOR pathway
4	*FGFR1* amp; *EGFR* amp; *IGF1R* amp; *ERBB2/3/4* mut; *BRAF* amp/mut; *KRAS* amp/mut; *HRAS* mut; *DUSP4* del	RAS/RAF/MEK pathway
5	*RB1* del; *CDK6* amp; *CCND1/2* amp	Cell-cycle checkpoints
6	*JAK2* amp	IL-6/JAK/STAT pathway
7	*Notch1/2/3/4* over-exp, *JAG1/2*, *JAG2*, *DLL4*	Notch pathway
8	*GLI1*, *Zeb1/2*, *Snail1/2*, *Twist 1/2*, *FOXM1*, *FOXC1/2* over-exp	Hedgehog signaling

Amp, gene amplification; del, gene deletion; mut, gene mutation; over-exp, over-expression.

**Table 2:** Genes whose mutations occur in TNBC and are not found in non-TNBC and the pathways of these genes according to TCGA database.

Gene	Pathway
*NOL6-9p13.3*	Major pathway of rRNA processing in the nucleolus and cytosol; rRNA modification in the nucleus and cytosol
*FAT3-11q14.3*	Wnt signaling pathway; cadherin signaling pathway
*AHNAK-11q12.3*	EGFR1
*BRCA1-17q21.31*	TP53 regulates transcription of DNA repair genes; processing of DNA double-strand break ends; ATM pathway; presynaptic phase of homologous DNA pairing and strand exchange; homologous DNA pairing and strand exchange; G2/M DNA damage checkpoint; generic transcription pathway
*CACNA1B-9q34.3*	Presynaptic depolarization and calcium channel opening
*F5-1q24.2*	Common pathway of fibrin clot formation; regulation of IGF activity by IGFBP; post-translational protein phosphorylation
*FLG2-1q21.3*	Neutrophil degranulation
*RNF213-17q25.3*	Antigen processing: ubiquitination and proteasome degradation
*ABCA9-17q24.2*	ABC transporters in lipid homeostasis; ABC-family proteins mediated transport
*DNAH5-5p15.2*	Huntington disease
*LAMA1-18p11.31*	MET activates PTK2 signaling; laminin interactions; L1CAM interactions
*PLXNA2-1q32.2*	SEMA3A-plexin repulsion signaling by inhibiting integrin adhesion; sema3A PAK-dependent axon repulsion
*VWF-12p13.31*	GP1b-IX-V activation signaling; integrin alphaIIb beta3 signaling; MAP2K and MAPK activation; signaling by high-kinase activity BRAF mutants
*DYNC2H1-11q22.3*	Huntington disease; hedgehog ‘off’ state
*PCNT-21q22.3*	AURKA activation by TPX2; regulation of PLK1 activity at G2/M transition
*SALL1-16q12.1*	POU5F1 (OCT4), SOX2, NANOG activate genes related to proliferation; transcriptional regulation of pluripotent stem cells; regulation of PTEN gene transcription; PI3K/AKT signaling
*TG-8q24.22*	Thyroid hormone synthesis; interleukin-6 signaling; constitutive signaling by AKT1 E17K in cancer; IL-6-type cytokine receptor ligand interactions; signaling by EGFR; signaling by ERBB4

## Data Availability

The data presented in this study are openly available in TCGA and Metabric databases.

## Supplementary Materials

The following are available online at https://www.mdpi.com/article/10.3390/cancers13215348/s1, Table S1: TNBC signaling cell pathways according to the TCGA database, Table S2: non-TNBC signaling cell pathways and common signaling pathways for TNBC and non-TNBC according TCGA database.
