# Supplementary material for: Molecular-Genetic Portrait of Breast Cancer with Triple Negative Phenotype
Source: Cancers (Basel). 2021 Oct 26;13(21):5348. doi: 10.3390/cancers13215348 (PMC8582512; doi:10.3390/cancers13215348)
Supplement: Supplementary file 1 [file cancers-13-05348-s001.zip › cancers-1432277-supplementary.pdf]

cancers

MDPI

Table S1. TNBC signaling cell pathways according to the TCGA database.

Pathway TNBC				
22q11.2 copy number variation syndrome	Dengue-2 interactions with blood clotting cascade	Focal adhesion: PI3K-Akt-mTOR-signaling pathway	Malignant pleural mesothelioma	Sandbox pathway test
3q29 copy number variation syndrome	Dengue-2 interactions with complement and coagulation cascades	GDNF/RET signaling axis	MAPK signaling pathway	Small cell lung cancer
Alpha 6 beta 4 signaling pathway	Development of ureteric collection system	Genes related to primary cilium development (based on CRISPR)	Meiotic recombination	SMC1/SMC3 role in DNA damage - Cornelia de Lange Syndrome
Androgen receptor signaling pathway	DNA damage response	Glioblastoma signaling pathways	Meiotic synapsis	Spinal cord injury
ATM signaling pathway	DNA Double Strand Break Response	Hepatitis C and hepatocellular carcinoma	miRNA regulation of DNA damage response	SUMOylation of DNA damage response and repair proteins
Blood clotting cascade	DNA IR-damage and cellular response via ATR	Homologous recombination	Nucleotide excision repair in xeroderma pigmentosum	Synaptic vesicle pathway
Breast cancer pathway	DNA IR-double strand breaks and cellular response via ATM	Integrated breast cancer pathway	Olfactory bulb development and olfactory learning	Thermogenesis
Calcium regulation in cardiac cells	DNA repair pathways, full network	Intraflagellar transport proteins binding to dynein	Pathways affected in adenoid cystic carcinoma	Thyroid hormones production and peripheral downstream signaling effects
Ciliopathies	Ectoderm differentiation	Joubert syndrome	PI3K-Akt signaling pathway	Transcriptional regulation by E2F6
Complement and coagulation cascades	Focal adhesion	Lipid particles composition	POU5F1 (OCT4), SOX2, NANOG activate genes related to proliferation	Vasopressin-regulated water reabsorption

Table S2. non-TNBC signaling cell pathways and common signaling pathways for TNBC and non-TNBC according TCGA database.

Sammary Pathway	Pathway non-TNBC					
Cell-type dependent selectivity of CCK2R signaling	Oncostatin M signaling pathway	Insulin signaling	Brain-derived neurotrophic factor (BDNF) signaling pathway	Neural crest cell migration in cancer	Complement system in neuronal development and plasticity	Small cell lung cancer
Pathways affected in adenoid cystic carcinoma	DNA damage response	Endometrial cancer	MAPK Signaling Pathway	Focal adhesion: PI3K-Akt-mTOR-signaling pathway	TP53 Regulates Metabolic Genes	Toll-like receptor signaling pathway
16p11.2 distal deletion syndrome	Melanoma	Pathways affected in adenoid cystic carcinoma	Regulation of toll-like receptor signaling pathway	miRNA regulation of prostate cancer signaling pathways	Transcriptional regulation of white adipocyte differentiation	Elabela signaling pathway
16p11.2 proximal deletion syndrome	Non-small cell lung cancer	Melanoma	Breast cancer pathway	Envelope proteins and their potential roles in EDMD physiopathology	Senescence and autophagy in cancer	Somatroph axis (GH) and its relationship to dietary restriction and aging

Ultraconserved region 339 modulation of tumor suppressor microRNAs in cancer	RAC1/PAK1/p38/MMP2 pathway	Head and neck squamous cell carcinoma	Calcium regulation in cardiac cells	Mechanoregulation and pathology of YAP/TAZ via Hippo and non-Hippo mechanisms	Circadian Clock	Androgen receptor network in prostate cancer
Sandbox pathway test	Chromosomal and microsatellite instability in colorectal cancer	Malignant pleural mesothelioma	Cell-type dependent selectivity of CCK2R signaling	Microtubule cytoskeleton regulation	Embryonic stem cell pluripotency pathways	NR1D1 (REV-ERBA) represses gene expression
Calcium regulation in cardiac cells	Arrhythmogenic right ventricular cardiomyopathy	Glioblastoma signaling pathways	Transcriptional cascade regulating adipogenesis	MyD88 cascade initiated on plasma membrane	DDX58/IFIH1-mediated induction of interferon-alpha/beta	Gastrin signaling pathway
Myometrial relaxation and contraction pathways	Head and neck squamous cell carcinoma	Phosphoinositides metabolism	MFAP5-mediated ovarian cancer cell motility and invasiveness	Thymic stromal lymphopoietin (TSLP) signaling pathway	Osteoblast differentiation and related diseases	Adipogenesis
MicroRNA network associated with chronic lymphocytic leukemia	Regulation of TP53 Expression and Degradation	DNA damage response (only ATM dependent)	EGF/EGFR signaling pathway	Photodynamic therapy-induced AP-1 survival signaling.	Fragile X syndrome	Dual hijack model of Vif in HIV infection
Mammary gland development pathway - Involution (Stage 4 of 4)	Meiotic synapsis	Angiopoietin-like protein 8 regulatory pathway	Integrated breast cancer pathway	Vitamin B12 metabolism	Signaling by FGFR3	Mammary gland development pathway - Involution (Stage 4 of 4)
TCA cycle in senescence	DNA IR-damage and cellular response via ATR	PI3K-AKT-mTOR signaling pathway and therapeutic opportunities	H19 action Rb-E2F1 signaling and CDK-Beta-catenin activity	Netrin-UNC5B signaling pathway	Extracellular matrix organization	Hair follicle development: organogenesis - part 2 of 3
Glycolysis in senescence	Glioblastoma signaling pathways	Ovarian infertility	Cancer immunotherapy by CTLA4 blockade	PI3K-Akt signaling pathway	TGF-beta signaling pathway	Overview of interferons-mediated signaling pathway
lncRNA-mediated mechanisms of therapeutic resistance	Apoptosis	MAPK cascade	Familial hyperlipidemia type 2	Hepatitis C and hepatocellular carcinoma	Measles virus infection	Alzheimer's disease
Apoptosis modulation and signaling	Pancreatic adenocarcinoma pathway	Hepatocyte growth factor receptor signaling	MAP3K1 role in promoting and blocking gonadal determination	IL-4 signaling pathway	Ebola virus pathway in host	G13 signaling pathway
Hereditary leiomyomatosis and renal cell carcinoma pathway	Retinoblastoma gene in cancer	p38 MAPK signaling pathway	Sex development	MyD88 dependent cascade initiated on endosome	Transcriptional regulation by MECP2	Kisspeptin/kisspeptin receptor system in the ovary
Effect of progerin on genes involved in Hutchinson-Gilford progeria syndrome	Small cell lung cancer	Hepatitis B infection	Mammary gland development pathway - Embryonic development (Stage 1 of 4)	Interferon type I signaling pathways	Ectoderm differentiation	Neural crest cell migration during development
Metastatic brain tumor	miRNA regulation of DNA damage response	Factors and pathways affecting insulin-like growth factor (IGF1)-Akt signaling	Overview of perturbations to host-cell autophagy, induced by distinct proteins of SARS-CoV-2	MET in type 1 papillary renal cell carcinoma	Signaling by FGFR1	Bladder cancer

NAD metabolism in oncogene-induced senescence and mitochondrial dysfunction-associated senescence	Wnt signaling pathway and pluripotency	Striated muscle contraction pathway	Apoptosis modulation by HSP70	Novel intracellular components of RIG-I-like receptor (RLR) pathway	Activation of anterior HOX genes in hindbrain development during early embryogenesis	Microglia pathogen phagocytosis pathway
Gastric cancer network 2	DNA damage response (only ATM dependent)	Epithelial to mesenchymal transition in colorectal cancer	BMP signaling in eyelid development	Development of ureteric collection system	Male infertility	Fc epsilon receptor (FCERI) signaling
miRNA regulation of p53 pathway in prostate cancer	Burn wound healing	mBDNF and proBDNF regulation of GABA neurotransmission	Hematopoietic stem cell gene regulation by GABP alpha/beta complex	MyD88:MAL(TIRAP) cascade initiated on plasma membrane	Pre-NOTCH Expression and Processing	IL-6 signaling pathway
Disruption of postsynaptic signaling by CNV	Senescence and autophagy in cancer	Host-pathogen interaction of human coronaviruses - MAPK signaling	Focal adhesion	Lung fibrosis	Regulation of actin cytoskeleton	T-cell receptor (TCR) signaling pathway
TP53 network	Spinal cord injury	Oxidative damage response	Circadian rhythm genes	Angiogenesis overview	22q11.2 copy number variation syndrome	Hippo-Merlin signaling dysregulation
Fluoropyrimidine activity	TGF-beta signaling pathway	Fas ligand pathway and stress induction of heat shock proteins	PKC-gamma calcium signaling pathway in ataxia	Thyroid stimulating hormone (TSH) signaling pathway	Oxidative Stress Induced Senescence	Transcriptional regulation of granulopoiesis
Photodynamic therapy-induced HIF-1 survival signaling	Measles virus infection	Translation inhibitors in chronically activated PDGFRA cells	Vitamin D in inflammatory diseases	Folate metabolism	Mesodermal commitment pathway	Acute viral myocarditis
Amyotrophic lateral sclerosis (ALS)	Ectoderm differentiation	Rett syndrome causing genes	Extracellular vesicles in the crosstalk of cardiac cells	Non-small cell lung cancer	Nonalcoholic fatty liver disease	PI Metabolism
Striated muscle contraction pathway	Endoderm differentiation	Synaptic signaling pathways associated with autism spectrum disorder	GDNF/RET signaling axis	Pathogenic Escherichia coli infection	Myometrial relaxation and contraction pathways	Hippo signaling regulation pathways
Bladder cancer	MAPK Signaling Pathway	Endothelin pathway	IL1 and megakaryocytes in obesity	Arrhythmogenic right ventricular cardiomyopathy	Orexin receptor pathway	Neural crest differentiation
Ferroptosis	22q11.2 copy number variation syndrome	Copper homeostasis	Kallmann syndrome	p53 transcriptional gene network	miR-targeted genes in squamous cell	Wnt signaling pathway and pluripotency
ATM signaling pathway	Breast cancer pathway	IL-1 signaling pathway	Regulation of RUNX1 Expression and Activity	Prolactin signaling pathway	Sudden infant death syndrome (SIDS) susceptibility pathways	MicroRNAs in cardiomyocyte hypertrophy
miRNA regulation of prostate cancer signaling pathways	Mesodermal commitment pathway	Pre-implantation embryo	PDGFR-beta pathway	Leptin signaling pathway	Chemokine signaling pathway	JAK/STAT pathway
Envelope proteins and their potential roles in EDMD physiopathology	Sudden infant death syndrome (SIDS) susceptibility pathways	Notch signaling pathway (Netpath)	Statin inhibition of cholesterol production	16p11.2 distal deletion syndrome	PTEN Regulation	miR-targeted genes in epithelium

SUMOylation of transcription factors	Epithelial to mesenchymal transition in colorectal cancer	CCL18 signaling pathway	T cell receptor and co-stimulatory signaling	16p11.2 proximal deletion syndrome	Ciliopathies	miR-targeted genes in muscle cell
Photodynamic therapy-induced AP-1 survival signaling.	Circadian rhythm genes	Th17 cell differentiation pathway	Uptake and function of anthrax toxins	Meiotic synapsis	Ras signaling	Deubiquitination
Transcriptional regulation by VENTX	IL-18 signaling pathway	MECP2 and associated Rett syndrome	Development and heterogeneity of the ILC family	Clear cell renal cell carcinoma pathways	Signaling by FGFR2	miR-targeted genes in lymphocytes
Copper homeostasis	PI3K-Akt signaling pathway	EGFR tyrosine kinase inhibitor resistance	miRNA regulation of p53 pathway in prostate cancer	Transport of bile salts and organic acids, metal ions and amine compounds	PIP3 activates AKT signaling	A modular map of Bradykinin signaling network
Netrin-UNC5B signaling pathway	miR-targeted genes in epithelium	Apoptosis	White fat cell differentiation	VEGFA-VEGFR2 signaling pathway	TCR signaling	Apelin receptor signaling pathway
Hepatitis C and hepatocellular carcinoma	miR-targeted genes in muscle cell	Androgen receptor signaling pathway	Serotonin HTR1 group and FOS pathway	Pancreatic adenocarcinoma pathway	ESR-mediated signaling	AXL signaling pathway
DNA IR-double strand breaks and cellular response via ATM	miR-targeted genes in lymphocytes	ErbB signaling pathway	Signal transduction through IL1R	Selenium micronutrient network	Interleukin-4 and Interleukin-13 signaling	CAMKK2 pathway
Endometrial cancer	AXL signaling pathway	Hematopoietic stem cell differentiation	Familial partial lipodystrophy (FPLD)	Signaling by FGFR4	IL-18 signaling pathway	Molecular targets for treatment of pancreatic cancer
G1 to S cell cycle control	-	TNF-alpha signaling pathway	Kinase-mediated control of CRTC2 and HDAC4/5/7 subcellular localization and activity	Hair follicle development: cytodifferentiation - part 3 of 3	PodNet: protein-protein interactions in the podocyte	Non-genomic actions of 1,25 dihydroxyvitamin D3
